# Optimization of nursing staff standards in the perioperative settings of the IRCCS University Hospital of Bologna. An improvement project

**DOI:** 10.3389/fpubh.2025.1601290

**Published:** 2025-06-26

**Authors:** Chiara Cenacchi, Martina Giusti, Angela Peghetti, Silvio Quirini, Francesco Tinelli, Sabina Giorgi, Salvatore Mineo, Manuela De Rosa, Stefano Durante

**Affiliations:** ^1^Departement of Healthcare Professions, IRCCS Azienda Ospedaliero-Universitaria di Bologna, Bologna, Italy; ^2^Department of Experimental and Clinical Medicine, University of Florence, Florence, Italy; ^3^Strategic Steering Commitee, Centro Studi SAPIS Foundation, Italian National Federation of Orders of Radiographers and Technical, Rehabilitation, and Prevention Health Professions Research Centre, Rome, Italy; ^4^SPIR, Professional Development and Research Implementation, IRCCS Azienda Ospedaliero-Universitaria di Bologna, Bologna, Italy

**Keywords:** nursing staff, perioperative settings, efficiency, effectiveness, reorganization

## Abstract

**Introduction:**

The organization of healthcare staff within operating block settings, which accommodate various surgical specialties, must consider the growing shortage of personnel and the need for resource optimization. For these reasons, we hypothesized that reorganizing the nursing staff could help reduce patient waiting lists, improve efficiency, and ensure patient safety.

**Methods:**

We conducted a review of the existing literature on Nursing Staff Standards in the Operating Rooms in relation to surgical procedures. IRCSS University Hospital of Bologna (Italy) hospital’s operating blocks (excepted pediatric surgical rooms) were chosen as experimental contexts due to the achievement of excellence according with the research mission of this hospital. Here, all implemented surgical procedures were classified and coded using ICD-9-CM codes. For each procedure, a reclassification process was applied based on the required nursing care intensity.

**Results:**

Results of literature review on Nursing Staff Standards in the Operating Rooms were applied, implementing a nursing roles’ reorganization. The reorganization moved from the incorporation of input from nurses and surgeons to identify areas for improvement and develop organizational solutions.

**Discussion:**

The reorganization process allowed for a redefinition of surgical schedules and staff allocation, leading to the reallocation of nursing units that were reassigned to support the opening of a recovery room.

## 1 Background

Several international studies (1–4) have already highlighted the benefits associated with the adoption of a new organizational model for staff allocation in the operating room. These studies highlight that staff allocation should consider not only the number of professionals, but also on their specific skills in relation to surgical procedures. Wright et al. (1) demonstrated that matching staff competencies to specific procedures can significantly reduce intervention time and improve postoperative outcomes. Nijkamp and Foran (2), in their study, found that the integration of adequately trained and specialized personnel into perioperative teams enhances patient safety and the quality of care. Amiri et al. (3) analyzed data from 21. OECD (Organization for Economic Co-operation and Development).

countries, confirming that an increase in staffing levels correlated with health professions’ competencies is associated with a reduction in surgical complications. Finally, Dall’Ora and Wright (4) explored the effects of flexible staffing, highlighting how the ability to adapt staff competencies to the specific needs of each operation can optimize both costs and clinical outcomes. These studies provide a solid foundation for revising organizational models in the operating room, proposing a more targeted and flexible approach that consider the specific needs of surgical procedures and staff competencies.

Such reorganization, therefore, if well designed, could lead to better utilization of staff and an improvement in outcomes due to better adherence to internationally validated clinical care protocols.

Some key principles for proper planning and reorganization of perioperative staff have been outlined in various international guidelines and studies (1, 4, 5, 6). These sources emphasize that effective perioperative staffing requires a structured approach that considers patient safety, workload distribution, and staff competencies. Specifically, the following principles are commonly recommended:

- Valuing staffing as a strategic resource, not merely a cost, recognizing its direct impact on care quality and safety.
- Remodeling the perioperative personnel budget according to changes in service demand and patient complexity.
- Ensuring safety for both patients and staff through adequate skill mix and workload balance.
- Defining organizational policies that set minimum staffing thresholds and quantify additional resources required for complex procedures.
- Involving nurses in decision-making processes related to perioperative organization and scheduling.
- Assessing patient characteristics and procedure complexity when planning staffing, possibly involving additional professionals when needed.
- Integrating staff organization strategies within broader quality improvement programs.

These principles are aligned with the recommendations of international bodies such as AORN and RNAO, which highlight the importance of adjusting staffing levels based on complexity and ensuring that each team includes nurses with advanced competencies. Moreover, recent literature has emphasized that such models not only improve patient outcomes but also contribute positively to staff satisfaction by promoting professional involvement, reducing work-related stress, and enhancing role clarity (1, 2). As a result, this approach fosters a more sustainable and supportive work environment in the perioperative setting.

Several international guidances (5, 6, 19), have been especially focused on the management of nursing staff during the perioperative phase. The following recommendations, that present only minimum differences, indeed, emphasize the importance of an adequate nurse–patient ratio to ensure safety and quality of care in the perioperative context. These particularly highlight that each procedure should include at least:

- a scrub nurse;
- a circulating nurse;
- additional support for complex situations or emergencies.

These recommendations promote planning based on local data and specific workload, considering the needs of the patients and the complexity of the surgical procedures. These also recommend that each team has nurses with advanced skills in the perioperative area, ensuring ongoing training. This includes assessing the specific skills needed for each type of surgery and ensuring that the staff has the appropriate qualifications.

Moreover, these recommendations support and suggest a personalized approach that actively involves nurses in resource planning, indicating two different approaches. The first is for more complex procedures or under general anesthesia that requires at least three nurses per room (1 anesthesia nurse, 1 scrub nurse, and 1 circulating nurse). The second is for less complex interventions, in which the number and composition of the nursing staff may vary. Therefore, it is recommended that the nursing staff be assigned based on the complexity of the surgical interventions and the conditions of the patient.

The Association of periOperative Registered Nurses, (6) especially empathizes the synergy between the operating room staff organization with continuity and the involvement of the staff handling preoperative care as crucial.

Registered Nurses’ Association of Ontario (5), instead, provides recommendations for implementing safe and effective staffing practices, with a focus on measuring and optimizing workload that must be adjusted according with perioperative dynamics and the complexity of the cases handled. In this way, it is promoted a sustainable work environment that supports both the nursing staff and patient outcomes.

Summarizing, the new proposed model affirms:

1. Adjustment of nursing staff based on the patient’s care complexity.
2. Implementation of the circulating nurse role.
3. Enhancement of professionals’ expertise and skills.

In Italy, the reorganization of nursing staff in the perioperative context has been implemented unevenly across the national territory, with regional policies incorporating some aspects of safe staffing but lacking uniform national standards aligned with international guidelines. Despite these challenges, the quality of care remains ensured. However, staff shortages and long waiting lists significantly impact surgical services. In 2023, surgical DRGs accounted for 27% of the most monitored DRGs nationwide, highlighting the central role of the perioperative pathway in hospital organization. Moreover, surgical processes absorb over 40% of hospital expenditures, further emphasizing the need for efficient resource management (7).

On the other side, the complexity of care in the operating room -defined by the intensity of nursing workload and quality of care- remains a key issue, reinforcing the necessity of structured nursing workload measurement and classification systems to support staff planning and ensure high-quality perioperative care (8). In fact, the organization of care based on intensity levels has been increasingly recognized worldwide as a fundamental model to optimize resource allocation and improve patient outcomes, including in the operating room setting. The same approach has been widely applied across regional healthcare system in Italy (9). This approach tailors staffing and perioperative care according to the complexity and intensity of each surgical procedure, ensuring that patients receive appropriate nursing support based on their specific needs. In the surgical context, care complexity is influenced by multiple factors, such as the type of intervention, patient comorbidities, and intraoperative risks, requiring a structured model to classify and allocate nursing resources effectively. In Italy, studies (10) have highlighted the importance of implementing nursing workload measurement systems to assess care intensity and support evidence-based staffing decisions. The SIPI model (Sistema Informativo delle Performance Infermieristiche) represents a structured framework for measuring nursing performance and classifying care intensity in various settings, including perioperative care. Integrating such models into operating room management can enhance efficiency, improve patient safety, and address the growing challenges related to staff shortages and high surgical demand.

## 2 Objectives

The main goal of the project is to apply the new model proposed in literature in the Italian context to optimize the efficiency of operating rooms through a more appropriate distribution of nursing staff maintaining patient safety and the quality of care provided, in accordance with current international guidelines. The secondary objective is to compare surgical complexity and nursing care complexity in order to verify the correspondence between the organizational standards required in international recommendations and in the Italian context.

The rationale is based on the idea that a better organization of resources, based on the actual complexity of the procedures, can reduce the downtime of operating rooms, improve the quality of care, and at the same time, optimize the allocation of nursing staff.

## 3 Materials and methods

The methodology used was a case study (11–14) of the IRCCS (Institutes of Hospitalization and Healthcare with Scientific Goal) University Hospital of Bologna as a specialized hospital facility serving as a regional reference, with several operating rooms distributed across five separate pavilions. Moreover, the own mission of this hospital is the continuous improvement of innovative organizational model to support better assistance pathways due to the presence of the Health Professions Directorate.

Here operating rooms cover a wide range of surgical specialties, including ophthalmology, urology, gynecology, cardiac surgery, general surgery, specialty surgeries, and transplants. Each surgical block has a specific internal organization separate from the other pavilions and has dedicated nursing staff.

Descriptive statistical techniques were adopted to identify correlations between surgical complexity and nursing care complexity. Qualitative methods were also used to better understand variations in the data and identify any necessary changes to the implementation of the organizational model.

**Figure 1:**
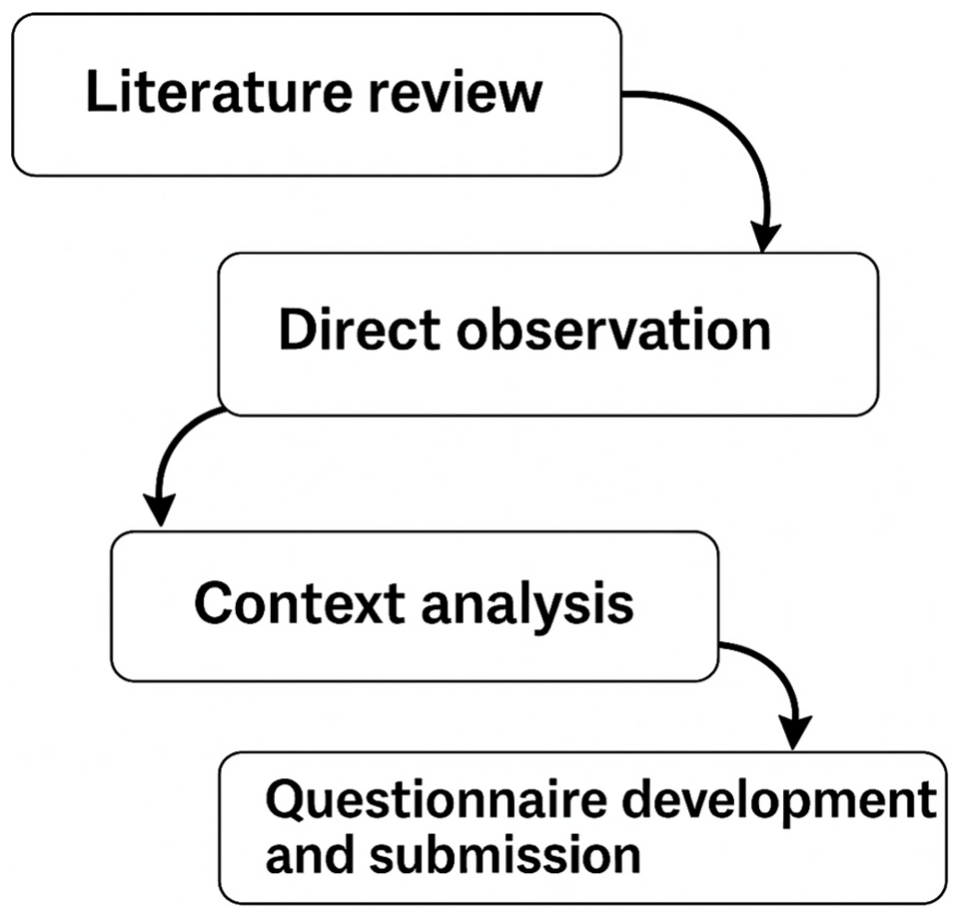
Four-phase process.

The reorganization project followed a structured four-phase process (summarized in Figure 1), as reported below:

- *Literature review.* The literature review was conducted using a systematic approach to identify, analyze, and synthesize relevant studies related to the reorganization of nursing staff and care intensity in the perioperative setting. A comprehensive search was performed across major scientific databases, including PubMed, CINAHL, Scopus, and Web of Science, to ensure the inclusion of peer-reviewed publications and international guidelines and policies related to safe staffing and care intensity classification.
- *Direct observation.* For 15 days, nursing activities were observed and recorded across all shifts and surgical specialties, regardless of the type of procedure. The observations were carried out under the supervision of an external clinical auditor, who routinely reviewed daily activities in the operating rooms to ensure consistency and objectivity in the data collection. Activities were categorized by role: anesthesia nurses, scrub nurses, and operating room nurses.
- *Context analysis.* Using ICD-9-CM codes, the surgical complexity of all surgical interventions performed in 2023 at our institute was analyzed. For the classification of the actual surgical complexity, the data collected were shared with the directors of the various surgical specialties. The data collected were analyzed to identify discrepancies between surgical complexity and nursing care complexity and to establish uniform criteria to implement the reorganization process.
- *Questionnaire development and submission.* Based on the observed and cataloged activities, three role-specific questionnaires were created (for anesthesia, scrub, and operating room nurses). Each item was rated on a 1–9 Likert scale (1–2 = very low complexity; 3–4 = low complexity; 5 = intermediate complexity; 6–7 = medium complexity; 8–9 = high complexity). The questionnaires were developed by the internal working group and validated with input from the external clinical auditor. They were administered to experienced nurses (minimum 10 years of OR service) across the five surgical blocks to classify the complexity of routine activities. An example is presented in Table 1.

**Table 1:** Questionnaire structure tailored in accordance with each profile.

Question no.	Anesthesia nurse	Scrub nurse	Operating room nurse
Question 1	Patient admission to the operating room	Surgical hand washing and dressing in a “sterile” environment	Recording of surgical times, professionals assigned to various roles, and entry/exit times
Question 2	Preoperative checklist verification	Preparation of the operating table	Collaboration with the scrub nurse in setting up the surgical field
Question 3	Preparation of induction and intraoperative drugs in collaboration with the anesthetist/resident	Surgical instrumentation for the procedure	Recording of high-cost medical devices
Question 4	Patient transfer and positioning on the operating table	Preparation of drains to be applied at the end of surgery	Supply of required materials during surgery
Question 5	Collaboration with the anesthetist for arterial catheterization and CVC placement	Removal of the surgical drape	Placement of urocontrol device or urinary catheter
Question 6	Assistance to the anesthetist during intubation	Surgical wound dressing	Verification of surgical instruments with the scrub nurse and counting of both instruments and gauze
Question 7	Administration of drugs	Verification of surgical instruments with the nurse operating room and counting of both instruments and gauze	Preparation of drains to be applied at the end of surgery
Question 8	Administration of cardioplegia drugs (where applicable)		Recording and sending of samples to microbiology
Question 9	Preparation of operating room equipment (TOF, BISS, Mechanical Ventilator, ECG Monitor, PA, SAO_2_/PLET, ETCO_2_, Blood Transfusion System, Masimo)		Collaboration with the anesthetist
Question 10	Assistance to the anesthetist during patient awakening		
Question 11	Recording of surgical times and roles of personnel involved		
Question 12	Verification of blood product requests in collaboration with the perfusionist (where applicable)		

This study did not require approval from an ethics committee or bioethics commission, as it was conducted as a Healthcare Organization Improvement Project. The Healthcare Professions Directorate directly authorized this study.

## 4 Results

The analysis of direct observation of nursing activities, conducted in the surgical blocks, reveals significant information about the distribution of resources and the organization of activities. The collected data were divided into 5 specific operational areas: Cardio/Thoracic/Vascular (CTV), General Surgery, Urology, Ophthalmology, and Gynecology, highlighting both overlaps and disparities in behaviors, as detailed in the following Table 2.

**Table 2:** Comparison of nursing activities in investigated settings.

Similarities	Differencies
*Nursing Roles and Responsibilities:* In each block, nurses perform similar tasks such as welcoming the patient, verifying preoperative checklists, preparing medications, and transferring and positioning the patient on the operating table. This indicates a standardization of nursing procedures across all departments. *Structuring of Operational Phases:* The sequence of preoperative, intraoperative, and postoperative activities follows a uniform model. In all departments, for example, scrub nurses are responsible for preparing and managing surgical instruments, while circulating nurses provide support in recording operative times and supplying materials. *Presence of the Anesthetist:* The anesthetist is always present in all departments during operations that require general and/or regional anesthesia. This role is of fundamental importance in ensuring patient safety during surgical procedures. *Multidisciplinary Collaboration:* In all blocks, there is a close collaboration among different professional figures (surgeons, anesthetists, nurses) to ensure the success of the procedures. Cooperation is particularly evident during the preparation and management of surgical instruments and materials.	*Number of Nurses:* The main difference concerns the number of nurses assigned to each operating room. For example, in Ophthalmology, there are only two nurses, unlike other departments which have three. This reflects the lesser complexity and invasiveness of ophthalmic procedures. *Specificity of Activities:* Some activities are specific to certain departments. For example, in the CTV (Cardio/Thoracic/Vascular) area, nurses are involved in preparing drugs for cardioplegia and managing complex equipment, such as hemodynamic monitoring devices. In Urology, greater attention is given to managing materials for catheterization. *Perfusion Technician:* The perfusion technician is predominantly present in the CTV block, where they are essential for complex Cardio-Thoracic-Vascular surgeries. This role is not mentioned in other departments, highlighting a difference in the type of support required. *Adaptation to Clinical Specifics:* Each department modifies its operative procedures based on the specific needs of its respective specialties. For example, Plate A/Plate B show a distribution of activities that reflects a greater variety of interventions, while in Ophthalmology and Urology, the procedures are more standardized and less complex.

A total of 1,671 ICD-9-CM codes, each corresponding to surgical procedures performed in 2023 at the IRCCS University Hospital of Bologna, were analyzed. Surgical complexity was classified into high, medium, or low, based on objective criteria such as expected duration of the procedure, type of anesthesia, invasiveness, and potential for intraoperative complications.

The classification was conducted in close collaboration with the surgical specialty directors. For each specialty, a panel composed of at least two senior surgeons independently assessed and discussed the procedures using a shared classification scheme. Discrepancies were resolved through consensus to ensure consistency and intersubjective reliability in assigning complexity levels.

Of these, 475 (28.2%) were found to correspond to high surgical complexity; 666 (40.3%) to medium complexity, and 530 (31.5%) to low complexity.

The activities were cataloged and weighted by nurses using the previously described questionnaires. The questionnaires were filled out on a single day in a context dedicated to this purpose, after explaining to the professionals the project’s objectives, the methods used, and the goals of the evaluation they were to express.

The results showed that most of the nursing activities in the operating room are of medium complexity, followed by those of low complexity, with few activities of high complexity.

Low complexity activities include surgical washing and sterile dressing, while medium complexity activities include the placement of arterial catheterization, checking the preoperative checklist, preparing medications and assisting during the awakening phase. High complexity activities include assisting the surgeon during the intraoperative phase and preparing operating room machinery such as respirators, electric scalpels, suction devices, etc. This classification reflects the responses provided by expert nurses in the questionnaire, based on their daily experience.

**Figure 2:**
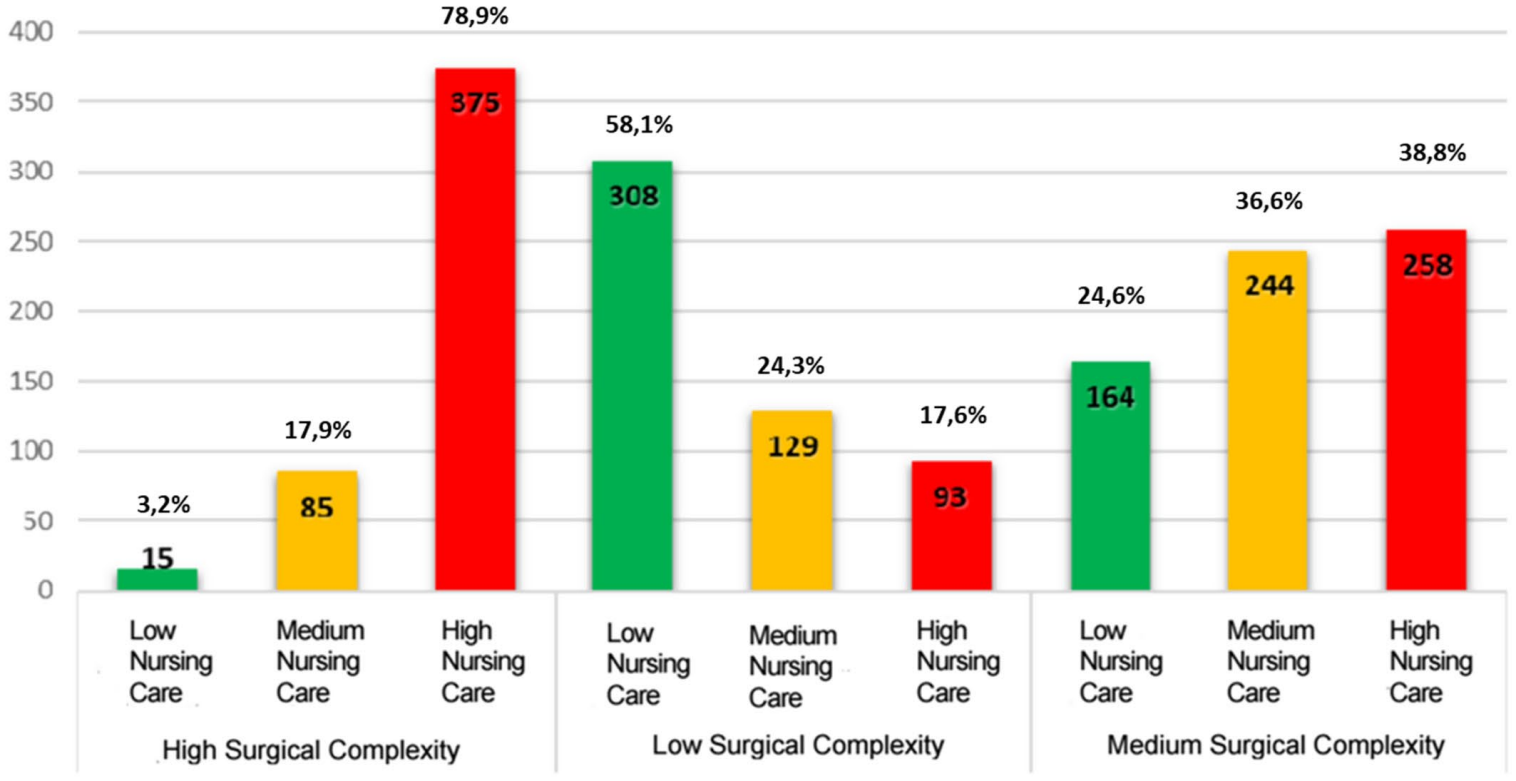
Comparison of surgical complexity vs. nursing care complexity.

From this, it can be deduced that anesthesia nurses perform the most complex tasks, while circulating nurses mainly handle activities of medium or low complexity (see Figure 2). The comparison between surgical complexity and nursing care complexity revealed discrepancies; the levels of complexity in surgery and nursing care required to manage the same procedure differ.

The chart shown in Figure 2 highlights a gap in nursing care complexity of 21% when compared with High Surgical Complexity, 63% for Medium Surgical Complexity, and 42% for Low Surgical Complexity.

In conclusion, the results of the comparative analysis show clear standardization of operative procedures across various surgical blocks and significant differences related to the clinical specificities of each area. The distribution of nursing responsibilities, the constant presence of the anesthetist, and multidisciplinary collaboration are uniform elements in all departments and ensure a consistent approach in managing operations. However, variations in the number of nurses, specificity of activities, and the presence of specialized roles such as the perfusion technician reflect the different operational needs of the specialties involved.

This analysis highlights the importance of a flexible and adaptable organization tailored to the peculiarities of each operating room to optimize the efficiency and safety of surgical procedures. From the data collected and the classification of the interventions, a reorganization was carried out that included the introduction of the “circulating nurse” as recommended by the AORN (6) and Botha (15).

**Figure 3:**
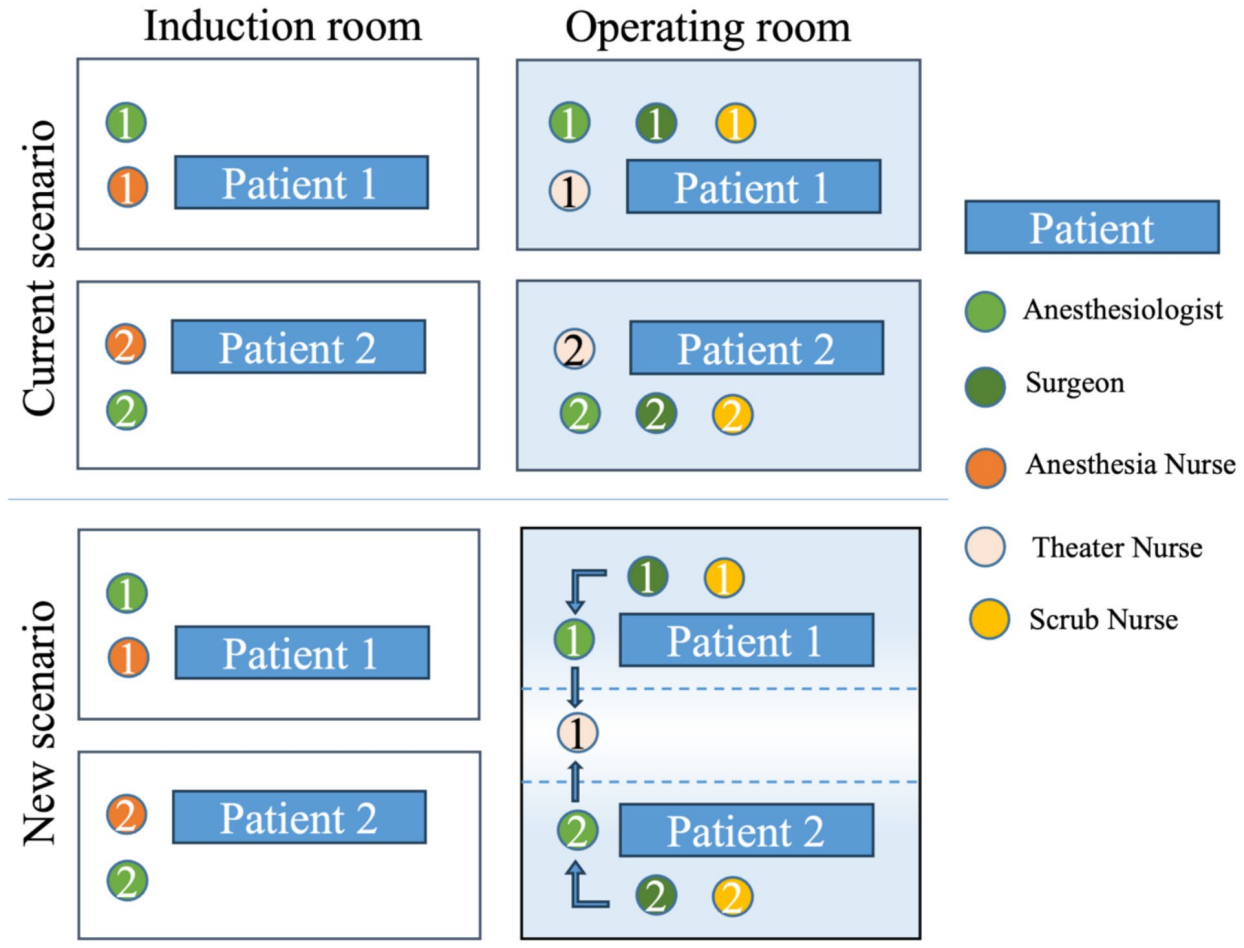
Comparison of current and the proposed new scenario in perioperative setting.

The organization of the operating rooms across various hospital blocks was redesigned, taking into account the different nursing care complexities required by the procedures, redefining the role and skills of the operating room nurse to ensure flexibility and adaptability of this figure according to needs. Regardless of the surgical block or type of surgery, interventions with high nursing care complexity require the presence of three nurses per operating room. For interventions of medium care intensity, two nurses per room were planned (1 anesthesia nurse and 1 scrub nurse) with a circulating nurse to cover two rooms. Finally, for interventions of low care intensity, two nurses per room (1 anesthesia nurse and 1 scrub nurse) are planned. The entire perioperative process workflow is currently being reorganized and includes the use of computer software for composing operative notes and defining the personnel dedicated to the different rooms (Figure 3).

## 5 Discussion

The analysis of the collected data has highlighted how the reorganization of nursing staff in the operating rooms of the IRCCS University Hospital of Bologna has had a significant impact on resource management, patient safety, and operational efficiency. The introduction of the circulating nurse role has enabled greater flexibility in staff allocation, improving the distribution of responsibilities based on the complexity of surgical procedures.

One key aspect that emerged from the study is the discrepancy between surgical complexity and nursing care complexity, with a gap of 21% for high-complexity procedures and 42% for low-complexity ones. This discrepancy underscores the need for more flexible and adaptable organizational models, allowing the number and role of nurses to be adjusted according to the actual care needs of each patient. Similar findings have been reported in studies evaluating perioperative staffing models, where inadequate nurse allocation was linked to prolonged surgical durations and increased postoperative complications (16, 17).

A comparison with the international scenario confirms that an approach based on the assessment of nursing care complexity enhances clinical outcomes and optimizes resource use. Previous studies (1, 2) have demonstrated that staff organization based on specific competencies reduces operative times and improves patient safety (1, 2). Additionally, research from Amiri et al. (3) and Dall’Ora and Wright (4) supports the idea that a higher level of staffing correlated with professional competencies is associated with a reduction in surgical complications and better perioperative care outcomes. Pasquer et al. (18) further reinforce that optimizing operating room processes through adequate nursing staff allocation leads to better efficiency and overall hospital cost reduction.

The implementation of workload measurement tools (19), such as the SIPI model, has allowed a more precise assessment of care needs, fostering data-driven planning.

While the SIPI model is particularly useful for assessing nursing care needs and optimizing staffing at a more localized level, the use of standardized methodologies for staffing needs assessment, such as the WISN (Workload Indicators of Staffing Need) model developed by the World Health Organization, could help manage human resources on a broader scale.

This tool allows for the calculation of the number of healthcare workers required to handle the workload of a specific healthcare facility and evaluates the workload demands placed on the staff in that facility (20).

WINS model has been applied in various healthcare settings to improve efficiency and equity in resource allocation (21–23).

Incorporating internationally validated models like WISN in the perioperative settings could enhance the precision of workforce planning and better align nursing staff with the real complexity of care.

Furthermore, guidelines provided by AORN (6) and the RNAO (5) emphasize the necessity of safe staffing ratios to enhance perioperative care, suggesting that at least three nurses (scrub nurse, circulating nurse, and anesthesia nurse) should be present for high-complexity procedures (19, 6).

The model implemented in this study not only aimed at improving care quality and workflow efficiency but also set the foundation for more strategic human resource planning. Applying this approach across all operating rooms could potentially result in measurable savings in terms of Full-Time Equivalents (FTEs), offering tangible benefits in workforce optimization. Considering not only direct staffing reductions but also indirect cost savings—such as decreased overtime, lower turnover, and improved staff productivity—would strengthen the economic validation of the model. Highlighting such data could further support the rationale for extending the model hospital-wide. This would enable the IRCCS University Hospital of Bologna to reallocate human resources more effectively across departments, ensuring that staffing aligns with actual care demands while maintaining patient safety and operational performance.

Despite these positive results, the study has some limitations. It was conducted in a single hospital setting, which limits the generalizability of the findings to other contexts. Moreover, the implementation of the organizational model required an adaptation period for staff, which could lead to variations in long-term results. Lastly, integrating new management and data collection tools remains a challenge that requires continuous monitoring to ensure the sustainability of the introduced changes.

## 6 Conclusion

This study has demonstrated that the reorganization of nursing staff in perioperative settings in accordance with nursing care complexity can enhance the efficiency and effectiveness of operating rooms, ensuring better resource utilization and increased patient safety. The introduction of the circulating nurse role and the adjustment of nursing staff numbers per operating room based on the type of procedure represent promising strategies for optimizing personnel management. However, the effectiveness of these interventions should be confirmed through further studies with objective outcome evaluations.

The findings highlight the need for a flexible approach to nursing resource planning, supported by workload measurement tools to ensure an equitable distribution of responsibilities. However, further multicenter studies are necessary to confirm the efficiency of the proposed model and assess its applicability in different hospitals.

Additionally, integrating digital systems for staff management and surgical scheduling could further enhance efficiency and improve the quality of perioperative care.

Despite these positive results, the study has some limitations. It was conducted in a single hospital, which restricts the generalizability of the findings to other contexts. Furthermore, the implementation of the proposed organizational model requires an adaptation period for staff, potentially leading to variations in long-term outcomes. Lastly, the integration of new management and data collection tools remains a challenge, necessitating continuous monitoring to ensure the sustainability of the introduced changes.

## Data Availability

The original contributions presented in the study are included in the article/supplementary material, further inquiries can be directed to the corresponding author.
